# Asymptomatic Malaria Cases and *Plasmodium* Species among BaAka Pygmies in Central Africa

**DOI:** 10.3390/pathogens13080682

**Published:** 2024-08-12

**Authors:** Daria Kołodziej, Wanesa Richert, Dariusz Świetlik, Krzysztof Korzeniewski

**Affiliations:** 1 Department of Epidemiology and Tropical Medicine, Military Institute of Medicine—National Research Institute, 128 Szaserów St., 04-141 Warsaw, Poland; dkolodziej@wim.mil.pl (D.K.); wrichert@wim.mil.pl (W.R.); 2 Department of Biostatistics and Neural Networks, Medical University of Gdańsk, 1 Dębinki St., 80-211 Gdańsk, Poland; dswietlik@gumed.edu.pl

**Keywords:** malaria, *Plasmodium*, mRDT, RT-PCR, BaAka Pygmies, Central African Republic

## Abstract

Malaria is a significant health problem in Africa, primarily due to the *Plasmodium falciparum* species, but this is not the only etiological factor responsible for malaria on the continent. The goal of the present research was to describe asymptomatic malaria cases and to identify *Plasmodium* species responsible for malaria in the BaAka Pygmies, inhabitants of the Central African Republic (CAR). Screening was realised in the period of August–September 2021 among 308 people, including 74 children and 234 adults reporting to a healthcare facility in Monasao (southwest CAR), an area inhabited by a semi-nomadic tribe of BaAka Pygmies. The study consisted of two phases. Phase I, which was conducted in Africa, consisted of performing malaria rapid diagnostic tests (mRDTs), taking haemoglobin measurements and collecting blood samples onto Whatman FTA cards for molecular diagnostics. Phase II, which was conducted in Poland, involved molecular tests (RT-PCR) to confirm or rule out malaria infections and to identify *Plasmodium* species responsible for the infections. mRDTs detected *Plasmodium* infections in 50.3% of children and 17.1% of adults participating in the study, whereas RT-PCR assays yielded positive results for 59.5% children and 28.6% adults. Molecular tests detected multiple *Plasmodium falciparum* infections but also three infections with *P. malariae*, three with *P. ovale* and one with *P. vivax.* The obtained results have confirmed numerous asymptomatic *Plasmodium* infections among the BaAka Pygmies. The rates of asymptomatic malaria cases in adults were twice as high as those in children, which may be indicative of the gradual acquisition of protective immunity with age. The study findings have also demonstrated that although most cases of malaria in Africa are caused by *P. falciparum*, three other species are also present in the region.

## 1. Introduction

Malaria caused by *Plasmodium falciparum* is a major health issue in tropical countries, particularly in Africa [1]. According to the WHO, *P. falciparum* is responsible for 99.7% of all malaria cases on the African continent and 100% in Sub-Saharan Africa [2]. Some malaria infections are asymptomatic, which means that patients show no malaria-related symptoms and have normal body temperatures although they are infected with *Plasmodium* parasites. Such naturally acquired protective immunity to malaria is achieved through continuous exposure to infections [3]. This type of immunity does not prevent re-infections but can reduce the risk of severe health outcomes [4]. The persistence of *P. falciparum* infections in semi-immune people, particularly older children, is a significant factor in malaria transmission. These asymptomatic carriers can harbour the parasites for extended periods, often over 18 months, without any clinical symptoms [5]. There are reports which suggest that asymptomatic carriers pose a bigger threat to healthy individuals than symptomatic patients, especially if they do not receive antimalarial treatment. In children, asymptomatic *Plasmodium* infections are often associated with complications such as anaemia, stunted growth or cognitive impairment [6]. Malaria continues to be a significant public challenge in many parts of Africa, especially among semi-nomadic communities living in Cameroon, Gabon, the CAR and the Democratic Republic of Congo [7].

The goal of the present research was to describe asymptomatic malaria cases and to identify *Plasmodium* species responsible for causing malaria in the BaAka Pygmies, inhabitants of the Central African Republic. 

## 2. Material and Methods

### 2.1. Study Group

Screening was realised in the period of August–September 2021 among 308 people, including 74 children (1–15 years) and 234 adults (16–76 years), both females and males, representing semi-nomadic BaAka Pygmies reporting to a healthcare facility in Monasao (southwest CAR). Monasao village, with a population of approximately 4000 people, lies at 510 m above sea level. The patients included in the study had to meet the following inclusion criteria: no malaria clinical symptoms, including temperature ≤ 37.5 °C. Patients who received any antimalarial drugs during the 4 weeks prior to the commencement of the study and those with difficult venous access were excluded from the research. The patients who met the above criteria were admitted to a local healthcare centre in Monasao, where they had blood samples taken, malaria rapid diagnostic tests (mRDT) performed, haemoglobin levels measured (with a portable Hb analyser) and their body weights recorded. During the next stage, blood samples which had been taken from the study participants were applied onto Whatman FTA cards for further diagnostics (molecular tests to be carried out during Phase II of the study). For paediatric patients, parental consent or consent from a child’s legal guardian was obtained. All medical procedures (also, the patient information leaflet, informed consent form and patient questionnaires) were carried out in English during Phase II of the study, which was conducted at the Department of Epidemiology and Tropical Medicine (DETM) in Poland and consisted of performing molecular tests (RT-PCR) to confirm or rule out malaria infections and to identify *Plasmodium* species responsible for the infections.

### 2.2. Malaria Screening

mRDTs (Pf/Pv/Pan; Beright, Hangzhou, China Alltest Biotech Co. Ldt.) for the detection of four different *Plasmodium* species (*P. falciparum*, *P. ovale*/*P. malariae* and *P. vivax*) were used. The tests were performed on venous blood coming from individuals manifesting no clinical symptoms of malaria (asymptomatic malaria was confirmed if the mRDT test result was positive). The mRDT sensitivity was 98.7%, and the specificity was 99.3% (according to the manufacturer) [8].

Molecular diagnostics by *RT-PCR*. Dried blood specimens (200–300 µL of venous blood) were collected in Africa on the Whatman FTA microcards [9], stored in tightly sealed foil packages with a moisture absorber and next transferred to the DETM in Poland. Four 2 mm discs were punched out from the Whatman FTA cards with the Harris Uni-Core punch, and then genetic material was isolated from the discs using the Sherlock AX Kit (A&A Biotechnology, Gdańsk, Poland) in line with the manufacturer’s instructions (a test for the manual isolation of genomic DNA working on the principle of nucleic acid absorption on ion-exchange membranes, combined with DNA precipitation with isopropanol) [10]. The isolated DNA was suspended in 100 µL of the TE buffer that was attached to the test kit and then stored in a freezer until further analyses. RT-PCR diagnostics were performed using the VIASURE Malaria RT-PCR Detection Kit for the qualitative detection of *Plasmodium falciparum*, *P. malariae*, *P. ovale* and *P. vivax* DNA [11]. RT-PCRs were run on an AriaMx RT-PCR system (Agilent Technologies, Santa Clara, California, USA), which was programmed according to following conditions of the thermal profiles (Table 1).

### 2.3. Haemoglobin Measurements

The haemoglobin concentration was measured in venous blood samples using a portable DiaSpect^TM^ analyser (EKF Diagnostics, Cardiff, United Kingdom). Normal haemoglobin levels and Hb thresholds indicative of anaemia were interpreted in line with the WHO criteria: mild anaemia (11.0–11.9 g/dL in women, 11.0–12.9 g/dL in men), moderate anaemia (8.0–10.9 g/dL) and severe anaemia (<8.0 g/dL).

### 2.4. Statistical Methods

All calculations were carried out with TIBCO Software Inc. (Palo Alto, CA, USA) (2017). The analyses used several different tests: the chi-square test (the difference in percentages), Student’s *t*-test or Mann–Whitney U test (differences in quantitative parameters between the two groups). To determine the impact of selected parameters on a positive test result for *Plasmodium*, univariate and multivariate regression was used. In multivariate analysis, only statistically significant parameters obtained in the univariate analysis were included. 

### 2.5. Ethical Approval

The research project was approved by the Committee on Bioethics at the Military Institute of Medicine, Warsaw, Poland (Decision No. 22/WIM/2020). Screening performed in Pygmy community was performed with the written consent of every patient and with the assistance of medical personnel working at the healthcare centre in Monasao.

## 3. Results

The study sample consisted of 308 asymptomatic patients of both sexes, including 74 children (24%) and 234 adults (76%), representing inhabitants of Monasao village (southwest CAR). Females accounted for 55.4% of the child population and 64.5% of the adult population. In children, the mean body weight was 19.3 (9.4) kg, and in adults it was 44.4 (6.4) kg. The mean body temperature was 36.57 (0.23) °C in children vs. 36.59 (0.25) °C in adults. The mean haemoglobin concentration was found to be 10.73 (1.60) g% in children vs. 11.85 (1.64) g% in adults, and it was significantly higher in adults (*p* < 0.0001).

Malaria rapid diagnostic tests (mRDTs) confirmed 54 infections (17.5%) with *Plasmodium falciparum* in 36 children and 18 adults and 5 infections (1.6%) with *P. falciparum* + Pan (*P. ovale*/*P. malariae*) in 1 child and 4 adults (Table 2). The proportion of positive mRDT test results for *Plasmodium falciparum* was significantly higher in children than in adults (*p* < 0.0001). 

The molecular tests (RT-PCR) performed on the same sample consisting of 308 patients during Phase II of the study identified 111 infections with *Plasmodium* species (36%), including 104 infections with *Plasmodium falciparum* (39 children and 65 adults), 3 infections with *P. malariae* (2 children and 1 adult), 3 infections with *P. ovale* (all cases in paediatric patients) and 1 infection with *P. vivax* (in an adult patient) (Table 3). The proportion of positive results for *Plasmodium falciparum* infections confirmed by molecular biology methods (RT-PCR) was significantly higher in children (*p* < 0.0001). Similarly, the proportion of *P. ovale* infections was also significantly higher in children (*p* = 0.0020). 

Molecular tests confirmed 49 of 59 positive results on mRDTs and detected another 62 *Plasmodium* infections (Table 1 and Table 2). RT-PCR tests detected three infections with *P. malariae*, three infections with *P. ovale* and one infection with *P. vivax*. Two patients were found to be co-infected with *P. falciparum* and *P. malariae*, two patients with *P. falciparum* and *P. ovale* and one patient with *P. falciparum* and *P. vivax.* Three positive test results on the mRDT for Pf + Pan were confirmed by RT-PCR, but only for *P. falciparum* infections. A total of 53 patients who were found to be infected with *Plasmodium* species (50% of patients with a positive RT-PCR test result) had haemoglobin levels < 11.0 g/dL. The mean haemoglobin concentration was 10.3 (1.3) g% in *Plasmodium*-infected individuals vs. 11.9 (1.6) g% in non-infected individuals. *Plasmodium*-infected patients had significantly lower haemoglobin concentrations (*p* < 0.0001). Similar findings were observed between the group of individuals with a positive mRDT vs. individuals with a negative mRDT: 11.0 g% vs. 11.9 g% (*p* < 0.0001), respectively. A correlation between haemoglobin concentration and body temperature was determined for *Plasmodium*-infected vs. non-infected individuals (separately for mRDTs and PCR assays). A statistically significant correlation between the two parameters was seen in patients with a positive PCR test results (correlation coefficient r = 0.23, *p* = 0.0180). Haemoglobin concentration was found to increase with increasing body temperature. The remaining correlations were not statistically significant. The study found no correlation between low levels of haemoglobin and the specific *Plasmodium* parasite responsible for infection. 

The univariate regression showed that the probability of mRDT(+) was higher among children at a younger age and with a lower body weight and lower haemoglobin concentration, whereas the multivariate analysis showed that a higher probability of mRDT(+) was associated with one parameter only, i.e., with lower haemoglobin concentration (Table 4).

The univariate regression showed that the probability of RT-PCR(+) was higher among children at a younger age and with a lower body weight and lower haemoglobin concentration, whereas the multivariate analysis showed that the probability of RT-PCR(+) was higher in individuals with lower haemoglobin concentrations and in children (Table 5).

## 4. Discussion

Malaria is a significant health problem in Africa and is responsible for 9% of all diseases detected on the continent. Most malaria cases that are diagnosed in European countries are imported from Africa by international travellers [2,12]. The goal of this study was to describe asymptomatic malaria in Central Africa and to confirm the occurrence of infections with various *Plasmodium* species using the example of a semi-nomadic BaAka Pigmies, inhabitants of the Dzanga Sangha National Park (southwest CAR), a region which is a popular tourist destination for many international travellers. According to the WHO, the number of malaria infections in Africa can be much higher than the official reports suggest, which is associated with numerous asymptomatic cases. Carriers of infection are rarely diagnosed or accurately treated, and this makes them serious reservoirs for malaria transmission to other people in their communities. Numerous asymptomatic malaria cases are a result of multiple re-infections and the gradual acquisition of protective immunity to malaria associated with an asymptomatic clinical course of the disease [13]. Younger children were found to be most at risk of acquiring malaria and developing malaria-related complications. Continuous exposure to *Plasmodium* species may lead to modifications in a child’s immune system and result in the acquisition of natural protective immunity; however, in some cases, an asymptomatic infection may progress into severe malaria and become life threatening to a child [14,15,16]. The present research has confirmed the occurrence of asymptomatic malaria in 59.7% of the seemingly healthy children and in 28.7% seemingly healthy adults. The obtained results are comparable to those of a scientific report by Maziarz et al. [17] conducted in rural communities in north Uganda, which found asymptomatic infections in 52.4% of the studied children. 

According to the WHO, *Plasmodium falciparum* is responsible for 99.7% of all malaria infections on the African continent [2]. However, in this study, mRDTs detected infections with non-*falciparum* species as well. RT-PCR tests were found to have a better diagnostic performance than rapid diagnostic tests, as RT-PCR assays detected more asymptomatic infections and identified more non-*falciparum* species. The authors’ previous study that had been conducted in the Central African Republic also demonstrated infections with various *Plasmodium* species [18]. The WHO reports on the prevalence of malaria and the distribution of *Plasmodium* species in Africa are an obstacle to the accurate diagnosis, treatment and elimination of malaria on the continent. The WHO-recommended interventions are primarily focused on the treatment of *P. falciparum* malaria, whereas the management of non-*falciparum* malaria requires different regimen combinations. The treatment of non-*falciparum* malaria is additionally complicated by numerous asymptomatic malaria cases in Africa. Asymptomatic infections can have serious long-term complications, especially in paediatric patients [19]. Asymptomatic malaria, which is found in all parts of Africa, is a major obstacle in the fight to eliminate the disease in the region, and asymptomatic carriers are an important reservoir for sustaining malaria transmission [20,21]. The findings of the present study have demonstrated that children are more at risk of acquiring malaria; the mean age of malaria-infected children was 5 years. The study has also shown that a decline in the number of malaria cases was associated with older age, which is the result of the acquisition of protective immunity through repeated exposure to pathogens in areas where malaria is endemic [3,22,23,24]. In older patients who have acquired immunity to malaria, the parasite density may fall below the limit of detection for mRDT, and RT-PCR assays will only be capable of detecting a trace of the parasite DNA. It was demonstrated that age appears to be a key factor influencing the persistence of the humoral response, as adults have a more intense and longer-lasting antibody response to *Plasmodium* antigens (antibodies provide protection against re-infection in the future) than children [25]. The present study was conducted on a sample of asymptomatic individuals who all had normal body temperatures. In children with asymptomatic malaria, haemoglobin concentrations ranged between 6.5 and 12.3 g/dL, and in healthy children, it ranged from 8.8 to 14.6 g/dL. The results have confirmed that asymptomatic malaria is associated with a drop in haemoglobin concentration and thus can lead to anaemia [26,27]. Diagnostic difficulties, high rates of asymptomatic cases, poor living conditions and limited access to healthcare are the main reasons why malaria is one of the biggest health issues in Africa. Asymptomatic malaria is associated with a number of serious complications which are particularly dangerous in children. The continuous development of molecular diagnostic techniques aims at reducing the costs of diagnostic tests. Such tests could be used for large-scale screening and for detecting a trace of DNA in even the smallest samples. The components of individual multiplex amplification should be adapted to the local epidemiological situation in order to maximise the effectiveness of the tests; however, the major obstacle in introducing large-scale population screening in Africa lies in the poor economic situation in the region. Specially adapted PCR tests performed by trained personnel could potentially facilitate a drastic reduction in asymptomatic malaria cases on the continent and could also be used to identify a specific parasitic species responsible for symptomatic malaria, especially in patients co-infected with different *Plasmodium* species. Such interventions would facilitate the effective treatment of malaria and therefore would be useful in the elimination of malaria in Africa [28]. 

## 5. Conclusions

The obtained results have confirmed numerous asymptomatic *Plasmodium* infections in BaAka Pygmies, especially in children. The main risk factors for malaria include poverty, poor sanitation, a nomadic lifestyle and living/staying in endemic areas of malaria. The rates of asymptomatic malaria cases were found to be two-fold higher in the adult population, which may be indicative of the gradual acquisition of immunity to malaria with age. The study has also demonstrated that although most malaria cases in Central Africa are caused by *P. falciparum*, three other *Plasmodium* species are also present in the region. 

## Figures and Tables

**Table 1:** Conditions of the thermal profile.

Cycles	Phase	Temperature	Time
1	Polymerase activation	95 °C	120 s
45	Denaturation	95 °C	10 s
	Annealing/extension (data collection)	60 °C	50 s

**Table 2:** Positive results from mRDT.

	mRDT		
	*P. falciparum* *n* (%)	*Pan* (*P. ovale*/*P. malariae*) *n* (%)	Total *n* (%)
Children	36 (48.6)	1 (1.4)	37 (50.0)
Adults	18 (7.7)	4 (1.7)	22 (9.4)

**Table 3:** Positive results from RT-PCR.

	RT-PCR				
	*P. falciparum* *n* (%)	*P. ovale* *n* (%)	*P. malariae* *n* (%)	*P. vivax* *n* (%)	Total *n* (%)
Children	39 (52.7)	3 (4.1)	2 (2.7)	0 (0.0)	44 (59.5)
Adults	65 (27.8)	0 (0.0)	1 (0.4)	1 (0.4)	67 (9.4)

**Table 4:** Univariate and multivariate regression of parameters associated with *Plasmodium* infection confirmed by mRDTs.

	Univariate		Multivariate	
Parameter	OR (95% CI)	*p*-Value	OR (95% CI)	*p*-Value
**Sex**				
Female	0.66 (0.37–1.17)	0.1549		
Male	01.52 (0.85–2.69)	0.1549		
**Children/adults**				
Children (1–15)	9.64 (5.12–18.14)	<0.0001	2.34 (0.49–11.12)	0.2858
Adults (>15)	0.10 (0.06–0.20)	<0.0001	0.43 (0.09–2.04)	0.2858
**Age**	0.94 (0.92–0.96)	<0.0001	0.98 (0.95–1.01)	0.2886
**Body weight**	0.92 (0.90–0.94)	<0.0001	0.98 (0.93–1.03)	0.3369
**Body temperature**	0.69 (0.21–2.23)	0.5331		
**Hb g/dL**	0.51 (0.41–0.64)	<0.0001	0.60 (047–0.77)	<0.0001

**Table 5:** Univariate and multivariate regression of parameters associated with *Plasmodium* infection confirmed by RT-PCR.

	Univariate		Multivariate	
Parameter	OR (95% CI)	*p*-Value	OR (95% CI)	*p*-Value
**Sex**				
Female	0.67 (0.41–1.08)	0.0987		
Male	1.50 (0.93–2.42)	0.0987		
**Children/adults**				
Children (1–15)	2.93 (1.71–5.02)	0.0001	4.00 (1.24–12.86)	0.0202
Adults (>15)	0.34 (0.20–0.58)	0.0001		
**Age**	0.98 (0.97–1.00)	0.0065	1.01 (0.99–1.03)	0.5353
**Body weight**	0.97 (0.95–0.99)	0.0006	1.02 (0.98–1.05)	0.3870
**Body temperature**	1.33 (0.50–3.54)	0.5637		
**Hb g/dL**	0.72 (0.62–0.84)	<0.0001	0.75 (0.63–0.88)	0.0006

## Data Availability

The data presented in this study are available on request from the corresponding author.
